# Clinical and pharmacogenomic predictors of survival in tamoxifen treated breast cancer female patients: a real-world study

**DOI:** 10.1186/s12885-025-14162-4

**Published:** 2025-06-01

**Authors:** Abdullah R. Al-Matrafi, Khaled F. Bedair, Sundararajan Srinivasan, Colin Palmer, Archie Campbell, Caroline Hayward, Ewan R. Pearson, Russell D. Petty

**Affiliations:** 1 Population Health and Genomics, School of Medicine, University of Dundee, Dundee, UK; 2 Department of Pharmacology, College of Clinical Pharmacy, Imam Abdulrahman Bin Faisal University, Dammam, Saudi Arabia; 3 Centre for Genomic and Experimental Medicine, Institute of Genetics & Cancer, University of Edinburgh, Western General Hospital, Edinburgh, EH4 2XU UK; 4 Usher Institute, University of Edinburgh, Edinburgh Bioquarter, 5-7 Little France Road, Edinburgh, EH16 4UX UK; 5 MRC Human Genetics Unit, Institute of Genetics and Cancer, University of Edinburgh, Western General Hospital, Edinburgh, EH4 2XU UK; 6 Molecular and Clinical Medicine, School of Medicine, University of Dundee, Dundee, UK

**Keywords:** Tamoxifen, Breast Cancer, *CYP2D6*, CYP2D6*4, *CYP2D6* inhibitors, SSRIs, Survival, Pharmacogenomics, Germline, Real-world

## Abstract

**Aim:**

To investigate the impact of tamoxifen dose, *CYP2D6* inhibitors, CYP2D6*4 genotype, and non-genetic parameters on the outcomes of tamoxifen treated female breast cancer patients.

**Method:**

We retrospectively included 3218 female patients who initiated tamoxifen following a diagnosis of breast cancer with long-term follow-up (median 7.5 years). A subgroup analysis of 303 genotyped patients with a median follow-up of 9.7 years was also conducted. The outcomes of interest were overall survival (OS) and breast-cancer-specific survival (BCS).

**Results:**

In the whole cohort, an additional 20 mg of tamoxifen during six-month duration was associated with a 1.6% reduction in all-cause mortality (HR: 0.984, 95% CI: 0.982–0.985, *P* < 0.001) and a 1.9% decrease in breast cancer mortality (HR: 0.981, 95% CI: 0.979–0.984, *P* < 0.001). In the genotyped subgroup, CYP2D6*4 heterozygotes had a 76% greater risk of all-cause mortality than *4 non-carriers (HR: 1.76, 95% CI: 1.07–2.9, *P* = 0.025). For breast cancer-specific mortality, CYP2D6*4 heterozygotes and homozygotes had increased risk by 3.7-fold (HR: 3.7, 95% CI: 1.32–10.6, *P* = 0.01) and 11.6-fold (HR: 11.6, 95% CI: 1.3–103.5, *P* = 0.03), respectively.

**Conclusion:**

Our study demonstrates that carriers of CYP2D6*4 have a higher risk of both all-cause and breast cancer-specific mortality and indicates that longer follow-up time may be crucial to determining impact. The shorter follow-up in previous studies may be a key reason for the conflicting results. A large real-world pharmacogenomic study with long-term follow-up is warranted to determine the impact of *CYP2D6* genotyping and its implications for clinical decision making.

**Supplementary Information:**

The online version contains supplementary material available at 10.1186/s12885-025-14162-4.

## Introduction

Invasive breast cancer was the most common cancer and the second leading cause of cancer-related mortality in UK females in 2017–2019 [1]. Globally, in 2020, breast cancer among females was the most common cancer, responsible for more than 2 million cases and 684,996 deaths [2]. One of the most effective treatments for breast cancer is tamoxifen, which is a selective oestrogen receptor modulator. Adjuvant tamoxifen, in oestrogen receptor positive early breast cancer patients, reduces breast cancer mortality [3, 4]. As a prodrug, tamoxifen is activated by the *CYP2D6* enzyme to the most abundant active form, endoxifen [5]. The metabolizer phenotype is determined by the highly polymorphic *CYP2D6* gene, which has an impact on endoxifen concentration and hence personalised tamoxifen dosing.

However, studies have reported conflicting results of *CYP2D6* phenotype on breast cancer outcomes, and accordingly, the role and implementation of *CYP2D6* genotyping in clinical decision-making remains controversial. A retrospective study of early breast cancer with a median 6.3-year follow-up found that carrying a reduced or non-functional *CYP2D6* allele was associated with worse tamoxifen outcomes [6]. However, another study with a median 6-year follow-up time, using “the Interpreting Breast International Group (BIG) 1–98” data, found no association between *CYP2D6* genotypes and tamoxifen outcomes [7]. In addition, many regulatory agencies, such as the FDA, EMA, HCSC, and PMDA, require genotyping for *CYP2D6* for different types of drugs, as summarised by PharmGKB [8]. For example, tetrabenazine, which is used for the treatment of Huntington’s disease, needs dose adjustment in accordance with CYP2D6 metaboliser phenotype. Since the vitality of *CYP2D6* genotype in altering enzyme activity is acknowledged for already used drugs, prior genotyping for tamoxifen could hold similar importance.

A recent systematic review and meta-analysis that included fifty-eight studies found that overall and disease-free survivals were associated with *CYP2D6* lower metabolizer status compared to normal [9]. However, they demonstrated that there were conflicts in the included studies. In addition, they found only one study reported the results of examining the interaction with selective serotonin reuptake inhibitors (SSRIs), indicating a gap in investigating concomitant drugs that can confound the association between *CYP2D6* genotypes and survival outcomes.

Thus, this study aimed to investigate, in a large real-world population with long follow-up, the impact of the tamoxifen dose coverage, CYP2D6*4 haplotype, and concomitant use of SSRIs, in addition to other non-genetic factors, on tamoxifen-treated breast cancer survival.

## Method

### Study design and population

In this retrospective observational cohort study, the population is comprised of female patients who lived in Tayside and Fife, Scotland, UK, and were diagnosed with invasive breast cancer between January 2000 and November 2021. Patients are eligible if they received tamoxifen within one year of diagnosis and had no more than a one-year gap between two consecutive prescriptions. The study protocol was approved by the Tayside Medical Science Centre (TASC) on April 26, 2022 (IRAS reference: 315039) and was conducted according to the STrengthening the Reporting of OBservational studies in Epidemiology (STROBE) guidelines [10].

### Clinical and genetic predictors

Using record linkage, data were extracted from prescribing data, demography data, the Scottish Cancer Registry (SMR06), and the national records of Scotland for death information. The invasive breast cancer incidences were identified using the “International Statistical Classification of Diseases and Related Health Problems 10” (ICD10) codes [11] from the SMR06. Prescribing data for other drugs was limited to the tamoxifen prescribing window, as the main interest here was to investigate the impact of drug-drug interactions.

To determine *CYP2D6* genotype, genetic data were obtained from three cohorts whose profiles were published before. These cohorts are: the Genetics of Diabetes Audit and Research in Tayside Scotland GoDARTS [12], the Genetics of the Scottish Health Research Register GoSHARE [13], and Generation Scotland: Scottish Family Health Study GS: SFHS [14]. The Health Informatics Centre (HIC) at the University of Dundee, UK, coordinated the data collection and standardisation, and it was made accessible for the researchers through a Trusted Research Environment (TRE).

### *CYP2D6* genotype

*CYP2D6* genotypes were categorized based on *4 haplotype using the additive model. CYP2D6*4 haplotype was chosen as it is the most common non-functional allele in Europeans [15]. In addition to *4, there are other non-functional haplotypes, such as *3, *5, and *6. The minor allele frequency (MAF) of *5 is 2.95% in Europeans [8], which is considered the second most common non-functional allele in this population. Moreover, the MAF for the representative SNPs of *3 (rs35742686) and *6 (rs5030655) is 1.3% and 0.167%, respectively, from a European reference population [16]. Due to technical limitations in our genotyping array data, we could not identify *5 or rs35742686, and given the rarity of rs5030655, we focused our analysis on the *4 (rs3892097) allele with a MAF of 19% [16].

### Statistical analysis

The follow-up started from tamoxifen initiation until the last patient observation. The tamoxifen start date was chosen over the patient’s diagnosis date to address survival time bias. Drug-drug and gene-drug interactions were examined using the extended Cox model and the Cox proportional hazard model, respectively. The results were presented as hazard ratios (HR) with their corresponding 95% confidence intervals (CI). All the analyses followed the intention-to-treat (ITT) approach. The study outcomes are overall survival (OS), which was the time between treatment and death from any cause, and breast cancer-specific survival (BCS), which was the time until breast cancer death. Censoring was applied when patients reached the study end date without experiencing the event. Various clinical covariates, in addition to the CYP2D6*4 genotype, were selected to investigate their association with the outcomes (see page 1 of the supplementary for more details). The proportional hazard assumption was examined using the global test for the Cox proportional hazard model. A chi-squared test was used to examine the difference between genotyped and non-genotyped cohorts, while for checking the difference between *4 genotypes, Fisher’s exact was used. All statistical analyses were performed using the R programming language [17].

## Results

### Patient characteristics

**Fig. 1:**
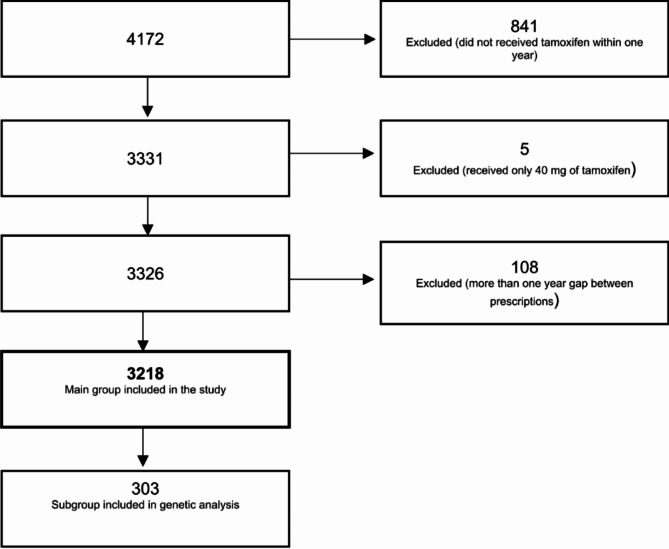
Study participants inclusion process

The study included 3,218 patients with invasive breast cancer, of whom 303 had genetic data (Fig. 1). The median age at treatment initiation was 60 (27–102) years, and the median follow-up of the study was 7.5 years. 673 individuals were administered at least one SSRI concomitantly with Tamoxifen. Among SSRIs, citalopram was observed to be the most frequently prescribed SSRI.

In the genotyped cohort, 95 patients were heterozygous for CYP2D6*4, 9 were homozygous, and the remaining 199 were non-carriers of this haplotype (see Table 2 in the supplementary for more details). The median age for this subpopulation was 60 (30–88) years, and the median follow-up time was 9.7 years.

A Chi-squared test comparing the genotyped and non-genotyped cohorts showed that being genotyped was associated with tumour size, lymph nodes, surgery, chemotherapy, and radiotherapy variables. For further details of the whole and genotyped cohorts, see Table 1 in the supplementary.

Across the genotyped subgroup, the *4 genotypes have no association with age, tumour size, grade, lymph nodes, oestrogen receptors, chronic kidney disease (CKD), letrozole, surgery, chemotherapy, or radiotherapy according to Fisher’s exact test (see Table 3 in the supplementary).

### Non-genetic predictors of tamoxifen breast cancer outcomes

#### Overall survival (OS)

In the univariate Cox model, all variables were associated with OS except for chemotherapy and letrozole (see Table 4 in the supplementary for more details).

**Fig. 2:**
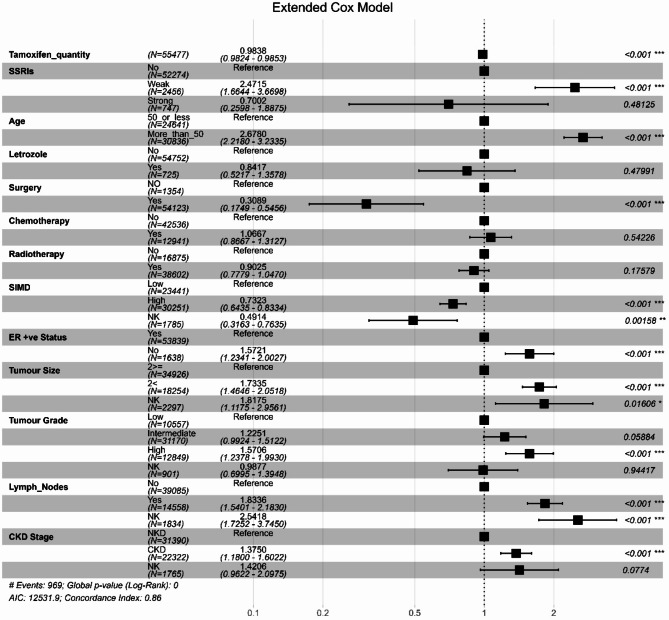
Forest Plot of the Hazard Ratios for covariates in the multivariable Cox Model for the Whole Cohort for Overall Survival Tamoxifen quantity: the total number of tamoxifen standard daily doses in the prior six months as a time-dependent variable. SSRIs: a time-dependent variable: weak = weak CYP2D6 inhibitors; strong = strong CYP2D6 inhibitors. SIMD: Scottish Index of Multiple Deprivation

In the multivariable extended Cox model (Fig. 2), a daily dose of 20 mg tamoxifen, the standard dose, was associated with a reduction in the risk of all-cause mortality by 1.6% (HR: 0.984, 95% CI: 0.982–0.985, *P* < 0.001) during the six-month interval. However, the use of weak *CYP2D6* inhibitors was correlated with a 2.5-fold increase in mortality risk compared to no SSRI (HR: 2.47, 95% CI: 1.66–3.67, *P* < 0.001) per six-month interval. Age greater than 50, tumour size, high tumour grade, lymph nodes, negative oestrogen status, and the presence of CKD were also associated with increased mortality risk, while surgery and the Scottish Index of Multiple Deprivation (SIMD) were correlated with a reduction in this risk.

#### Breast cancer specific survival (BCS)

In the univariate Cox model, only age, chronic kidney disease status, and letrozole treatment did not correlate with BCS (see Table 4 in the supplementary for more details).

**Fig. 3:**
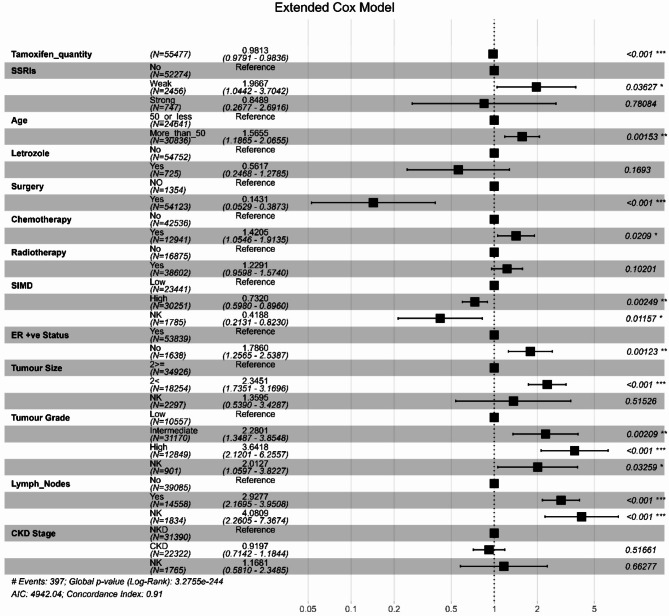
Forest Plot of hazard ratios for covariates in the multivariable Cox Model for the Whole Cohort for Breast Cancer specific Survival Tamoxifen quantity: the total number of tamoxifen standard daily doses in the prior six months as a time-dependent variable. SSRIs: a time-dependent variable: weak = weak CYP2D6 inhibitors; strong = strong CYP2D6 inhibitors. SIMD: Scottish Index of Multiple Deprivation

In keeping with the findings of the extended Cox model (Fig. 3), a reduction in breast cancer mortality risk by 1.9% was associated with each additional 20 mg tamoxifen per six-month interval (HR: 0.981, 95% CI: 0.979–0.984, *P* < 0.001). On the other hand, at each six-month interval, the use of weak CYP2D6 inhibitors was associated with around a one-fold increase in breast cancer mortality (HR: 1.97, 95% CI: 1.04–3.70, *P* = 0.04). In addition, age, chemotherapy, negative oestrogen receptor status, tumour size, worse tumour grade, and the presence of lymph nodes were associated with an increased risk. However, breast cancer-specific mortality was found to be reduced by surgery and SIMD.

### CYP2D6*4 genotype impact on survival

#### Overall survival (OS)

**Fig. 4:**
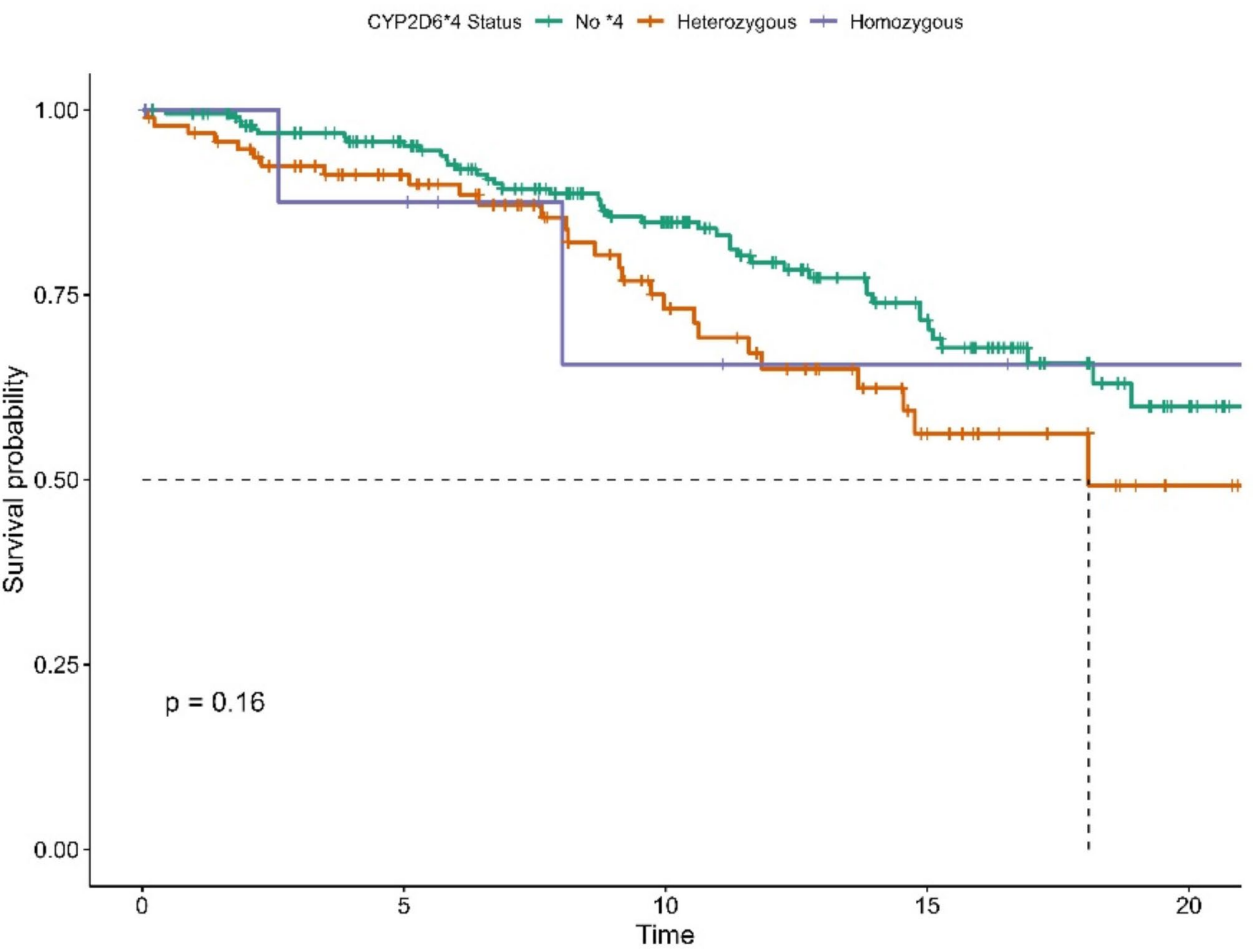
Kaplan-Meier Survival Curve for CYP2D6*4 Genotypes for OS

Survival curves, as illustrated in Fig. 4, for heterozygous carriers and non-carriers of *4 began to diverge around year eight. However, this difference was not statistically significant (Log-Rank test, *P* = 0.16). In the multivariable Cox model, there was a 76% increase in the risk of all-cause mortality associated with the heterozygous *4 genotype (HR: 1.76, 95% CI: 1.07–2.9, *P* = 0.025). For other variables, see Table 6 in the Supplementary.

#### Breast cancer specific survival (BCS)

**Fig. 5:**
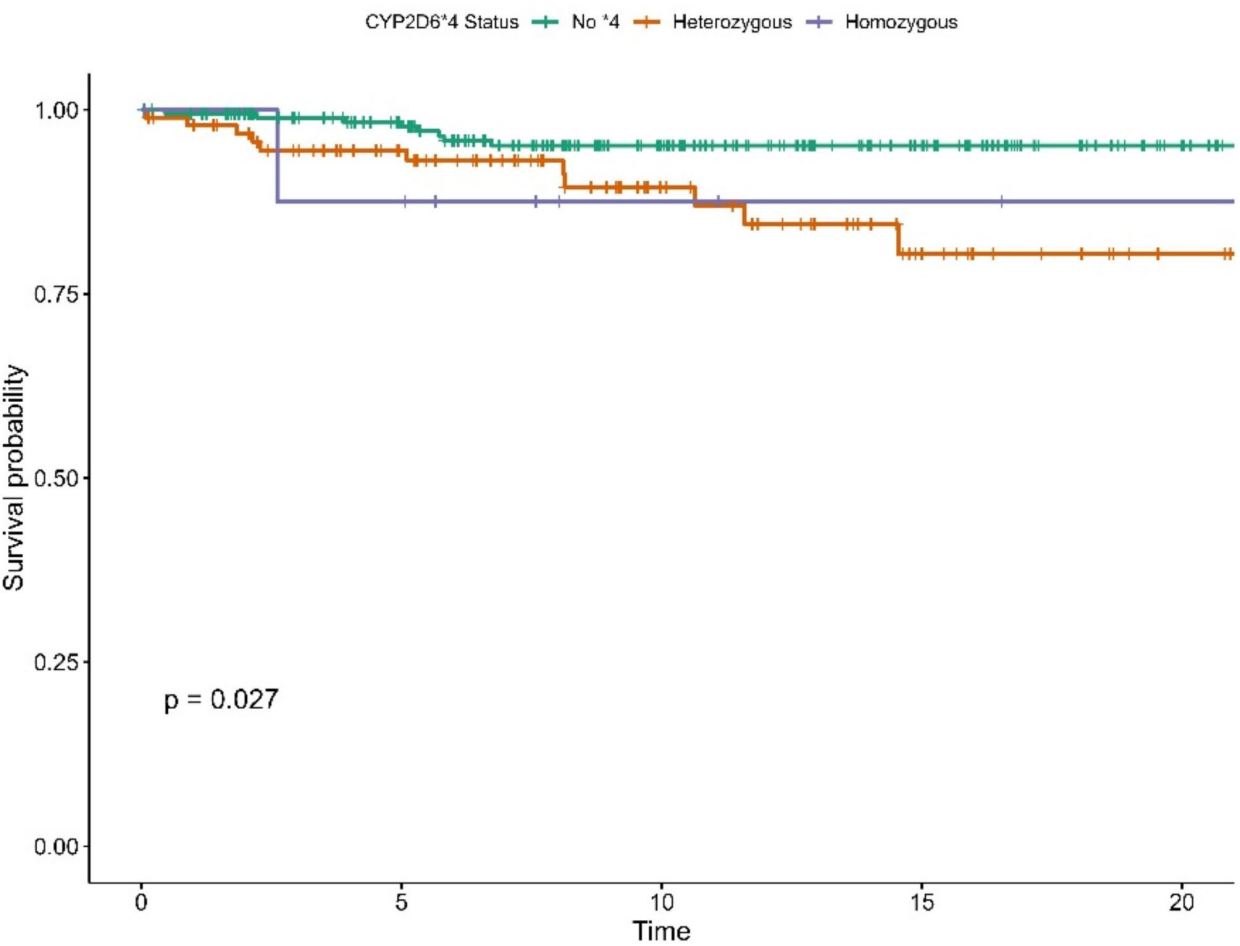
Kaplan-Meier Survival Curve for CYP2D6*4 Genotypes for BCS

In the univariable Cox model, the heterozygous *4 genotype was associated with a lower BCS (see Table 5 in the Supplementary). The survival curves stratified by genotypes showed a statistically significant difference (Log-Rank *P* = 0.027) and revealed that the increased risk associated with the *4 genotype became marked after 8 years of tamoxifen treatment, as illustrated in Fig. 5.

The multivariable analysis (see supplementary Table 6) showed that carrying the heterozygous *4 genotype was linked to a 3.7-fold higher risk of death from breast cancer (HR: 3.7, 95% CI: 1.32–10.6, *P* = 0.01). Despite the small number of events, the homozygous *4 genotype demonstrated a striking 11.6-fold increase in breast cancer death (HR: 11.6, 95% CI: 1.3–103.5, *P* = 0.03).

### Sensitivity analysis

#### Impact of COVID19

A sensitivity analysis was conducted to examine whether Coronavirus disease (COVID-19) had a confounding impact on the survival of the study population. All the previously shown analyses were repeated, with the end date of the observation period set at March 1st, 2020. The results were largely unchanged, indicating that the influence of COVID-19-related deaths on this cohort was negligible.

#### Immortal time bias

To investigate the impact of immortal time bias on survival, we conducted a sensitivity analysis by running the analysis on patients who genotyped before cancer diagnosis. 143 patients were included, and the univariate analysis revealed that OS and BCS had a similar direction of effect to those of the genotyped group. However, due to the limited number of events, conducting the multivariable analysis was not possible.

## Discussion

In this large-real-world observation study with long follow-up for tamoxifen use in females with breast cancer, we found that during each six-month intervals, a 20 mg standard daily dose of tamoxifen was associated with a 1.6% and 1.9% improvement in overall and breast cancer-specific survival, respectively. In addition, SSRIs with weak *CYP2D6* inhibition were associated with lower overall and breast cancer specific survival. We also found CYP2D6*4 carriers had 1.76- and 3.7-fold worsening of overall and breast cancer-specific survivals, respectively, while *4 homozygotes had a striking 11-fold increase in breast cancer-specific mortality.

In our study, we found that a standard 20 mg daily dose of tamoxifen over each six-month duration was associated with improving overall and breast cancer-specific survival. To the best of our knowledge, no study has investigated tamoxifen quantity in such time-dependent method. However, tamoxifen quantity varied over time, with at least 25% of six-month intervals having no recorded tamoxifen prescription (Q1 = 0), while 75% of intervals had up to 180 standard daily doses (Q3 = 180). There are studies investigating tamoxifen adherence in terms of prescription coverage that have found a link between adherence and improved survival. In a study conducted in Tayside, Scotland, researchers discovered that over half of females discontinued tamoxifen before the five-year mark, and they linked low adherence, defined as less than 80%, to all-cause mortality [18]. In addition, taking tamoxifen for five years is known to reduce breast cancer mortality in the first 15 years by one third, based on clinical trial data [19]. As tamoxifen quantity was modelled as a time-dependent variable, the hazard ratio does not represent a fixed effect over the entire follow-up time; instead, it reflects the dynamic change in the risk at every six-month time point. This highlights the need for caution when comparing the results with studies that used drug quantity as a time-fixed variable. Nonetheless, this study and clinical trial findings demonstrated the importance of tamoxifen dose and duration in improving patients’ survival.

We found that the concomitant use of SSRIs impact on survival outcomes was not fully consistent with the literature. The signal in our results is mainly driven by the weak *CYP2D6* inhibitor citalopram (data not shown), which might reflect an indication bias. In contrast, Abraham et al. showed that the use of *CYP2D6* inhibitors was not associated with either OS or BCS [20]. However, there were no details of how this analysis was performed, as they included different types of *CYP2D6* inhibitors. A different result was reported by Kelly et al. as they investigated concurrent intake of *CYP2D6* inhibitors with tamoxifen based on overlapping prescribing times, and they found only paroxetine use was linked with an increase in breast cancer and overall mortality [21]. Similarly to tamoxifen quantity, SSRI use was also modelled as a time-dependent variable, reflecting a dynamic change every six-month time point rather than a fixed risk over the whole follow-up time. These findings suggest that no conclusion can be drawn regarding SSRIs use, mainly because of the inconsistent designs of studies investigated the SSRIs interaction with tamoxifen.

Our findings that people with the reduced function CYP2D6*4 haplotype were at a greatly increased risk of all-cause and breast-cancer mortality agree with the Clinical Pharmacogenetics Implementation Consortium (CPIC) Guidelines for *CYP2D6* genotyping prior to tamoxifen treatment, which recommend the implementation of *CYP2D6* genotyping prior to tamoxifen treatment in breast cancer settings [22]. Nevertheless, numerous studies refute these results by revealing no correlation between *CYP2D6* genotypes and tamoxifen outcomes. A study, using “the Interpreting Breast International Group (BIG) 1–98” data, investigated tamoxifen against letrozole in early breast cancer settings and found no association between *CYP2D6* genotypes and tamoxifen outcomes [7]. One possible explanation for this inconsistency may be that their genotyped patients had a median follow-up time of 72 months, while in our study, follow-up was over 116.4 months for the genotyped subgroup, and this could enable a long-term drug-gene impact to become apparent. Additionally, the genotyping of tumour samples may also compromise these results, as a heterozygous individual—due to loss of heterozygosity (LOH) in tumour DNA—may be misinterpreted as homozygous, leading to the misclassification of metabolizer status as discussed by Stanton et al. [23]. In contrast to CPIC, our findings challenge the recommendations of the ESMO Clinical Practice Guidelines for Early Breast Cancer in 2020, which recommended against the use of *CYP2D6* to inform adjuvant tamoxifen decisions [24]. In the most updated version of these guidelines, there was no mention of *CYP2D6* genotyping [25], which still reflects the ESMO view of its lack of value in these settings.

Our study has many strengths, including the long follow-up time, which was 7.5 and 9.7 years for the whole and genotyped cohorts, respectively. In addition, the availability of the prescribing data enabled investigating the impact of concomitant drugs during tamoxifen treatment as well as tamoxifen quantity in a time-dependent fashion. Using blood samples as a source of genetic data is also an advantage for determining the germline genotype. Furthermore, we have thoroughly investigated the non-genetic variables, which we not reported in many studies. However, we acknowledge some weaknesses in our design, as using only *4 allele can lead to inaccurate *CYP2D6* genotype assignment. Missing genetic data for other important non-functional alleles like *3 and *5 can lead to wrongly combining normal metabolisers and poor metabolisers under one category. The retrospective observational nature of the study can also lead to errors in the data, such as missing data or entry errors. In addition, the small number of the genotyped subgroup, which represent 9.42%, makes it difficult to generalise genetic analysis results to the entire cohort. In the supplementary Table 1, we found that the genotyped and non-genotyped cohorts differed in tumour size, lymph nodes, surgery, chemotherapy, and radiotherapy variables, indicating that the genotyped subgroup is unrepresentative, possibly due to selection bias.

Our results suggest that tamoxifen treatment should be administered with greater caution based on the *CYP2D6* genotype of the patient. Close monitoring of concurrent drugs is warranted, given that patients are required to take the medication for a minimum of five years, a duration that can increase the risk of drug-drug interactions. We acknowledge that to establish the risk of CYP2D6*4 on tamoxifen outcomes, a large, randomised study is required with more than 10 years of follow-up. This could be undertaken using a pragmatic design, using remote decentralised methods and data linkage follow-up. Nevertheless, delivery of this would be very challenging and may be unfeasible. In this context, at present, females with breast cancer who are *CYP2D6* poor metabolizers should be considered for alternative treatments to tamoxifen; if none are available, the daily tamoxifen dose could be increased.

## Conclusion

In conclusion, tamoxifen quantity and CYP2D6*4 allele are important predictors of female breast cancer patients’ survival. We recommend that until a long follow-up randomized study clarifies the current controversy, for those with the poor metabolizer *CYP2D6* phenotype, consideration should be given to increasing the dose or switching to an alternative medication than tamoxifen.

## Electronic supplementary material

Below is the link to the electronic supplementary material.

Supplementary Material 1

## Data Availability

The data supporting the findings of this study are available upon reasonable request and with the necessary approvals via a controlled-access trusted research environment maintained by the Health Informatics Centre (HIC) at the University of Dundee, UK. https://www.dundee.ac.uk/hic.

## Abbreviations

OS: Overall survival
BCS: Breast-cancer-specific survival
ITT: Intention-to-treat
MAF: Minor allele frequency
HR: Hazard ratio
CI: Confidence intervals
Q1: First quartile
Q3: Third quartile
SIMD: Scottish Index of Multiple Deprivation
SSRIs: Selective serotonin reuptake inhibitors
CKD: Chronic kidney disease
COVID-19: Coronavirus disease
SMR06: Scottish Cancer Registry
ICD10: International Statistical Classification of Diseases and Related Health Problems 10
GoDARTS: Genetics of Diabetes Audit and Research in Tayside Scotland
GoSHARE: Genetics of the Scottish Health Research Register
GS:SFHS: Generation Scotland: Scottish Family Health Study
HIC: The Health Informatics Centre
TRE: Trusted Research Environment
CPIC: The Clinical Pharmacogenetics Implementation Consortium
FDA: The Food and Drug Administration
EMA: The European Medicines Agency
HCSC: The Health Canada/Santé Canada
PMDA: The Pharmaceuticals and Medical Devices Agency
