# Supplementary material for: Clinical and pharmacogenomic predictors of survival in tamoxifen treated breast cancer female patients: a real-world study
Source: BMC Cancer. 2025 Jun 1;25:974. doi: 10.1186/s12885-025-14162-4 (PMC12128510; doi:10.1186/s12885-025-14162-4)
Supplement: Supplementary file 1 — Supplementary Material 1 [file 12885_2025_14162_MOESM1_ESM.docx]

Explanatory Covariates

For survival analysis, many demographic and clinical characteristics were included as baseline covariates. Age was estimated at treatment start; to reduce outliers’ effect, it was divided into two categories by a 50-year limit. The Scottish Index of Multiple Deprivation (SIMD) was used to assess the patient's socioeconomic status, with levels 1 being the most deprived and 5 being the least. The SMID low category included levels 1, 2, and 3, to indicate areas with low socioeconomic status (highest deprivations), whereas the high category included 4 and 5 to indicate high socioeconomic status (lowest deprivation). Tamoxifen quantity was calculated for every six-month interval as a time- dependent variable according to the following equation:

$$T_{i}=\left( \frac{\sum_{j=1}^{ni} \left( Q_{\mathrm{ij}}\times S_{\mathrm{ij}} \right)}{20} \right)$$

Where:

i: index for the six-month interval

j: index for the prescription number within interval i

Ti: tamoxifen quantity which is the number of standard doses for prescription (j) within interval (i)

Qij: number of tablets for prescription (j) within interval (i).

Sij: tamoxifen strength (mg) for prescription (j) within interval (i).

ni: prescription counts within interval (i).

20: the standard tamoxifen daily strength for breast cancer.

We modified the model 6 method, which Abrahamowicz et al. illustrated(1), to calculate tamoxifen quantity. We calculated the standard dose equivalents in the past six months by multiplying the tablet count with strength and dividing the quantity by 20. Notably, a one-unit increase of tamoxifen quantity (Ti) corresponds to an additional 20 mg of tamoxifen per six-month interval. Covariates for tumour characteristics were size, grade, lymph nodes, and oestrogen receptor status. Tumour size was divided into three categories: =< 2 cm or > 2 cm. the tumour grade was categorised into low, intermediate, or high which reflect the level of undifferentiation, with high being the most severe grade. The lymph node variable was categorised as “Yes” or “No” to indicate if the lymph nodes were present or not, respectively. The classification of oestrogen receptor status was either positive or negative, with positive indicating the presence of detectable oestrogen receptors and negative indicating their absence or undetectable status. Normal kidney function was assigned to stage 1, while chronic kidney disease consisted of stages 2, 3, and 4. SSRIs, a time-dependent variable, were combined as weak (sertraline, citalopram, and escitalopram) and strong (paroxetine and fluoxetine) *CYP2D6* inhibitors. Letrozole, surgery, chemotherapy, and radiotherapy covariates are included as a categorical variable with “Yes” or “No” to indicate if the treatment was ever given or not, respectively. For radiotherapy and chemotherapy variables, patients who reported planned or unknown status in SMR06 were added to the “No” category. For the surgery variable, patients were assigned to the “Yes” category when they had a surgery date. The unknown category was used for missing values, except for surgery and chemotherapy, which were combined with the no category since no treatment information was most likely not given. In addition, in oestrogen receptor status, the unknown category was combined with the negative status since the numbers were too small. For radiotherapy, there were no missing values. All covariates are categorical except for the tamoxifen daily quantity, which is continuous.

Table 1: Study Population Characteristics and Chi-squared test

|  | **The whole cohort** | **The genotyped cohort** | **Chi squared test P-value**  **(Non-genotyped vs. genotyped cohort)** |
| --- | --- | --- | --- |
| Covariate | Median (range)/ No. of patients | Median (range)/ No. of patients |  |
| **Age** (years) | 60 (27 – 102) | 60 (30 – 88) | - |
|  | | | |
| **Age (categorical)** | | | |
| **≤55** | 1291 | 112 | 0.26 |
| **>55** | 1927 | 191 |  |
|  | | | |
| **Median follow-up time** (years) | 7.5 (0.005-21.8) | 9.7 (0.05 -21.4) |  |
|  | | | |
|  | | | |
| **Tumour size cm** | | | |
| ≤2 | 1913 | 293 | 0.005* |
| >2 | 1012 | 92 |  |
| Unknown | 293 | 13 |  |
|  | |  |  |
| **Tumour Grade** | |  |  |
| Low | 534 | 55 | 0.051 |
| Intermediate | 1843 | 181 |  |
| High | 722 | 64 |  |
| Unknown | 119 | 3 |  |
|  | | |  |
| **Lymph Nodes positive** | |  |  |
| No | 2139 | 237 | <0.001* |
| Yes | 821 | 55 |  |
| Unknown | 258 | 11 |  |
|  | | |  |
| **Oestrogen Receptors positive** | |  |  |
| No | 143 | 7 | 0.08 |
| Yes | 3075 | 296 |  |
|  | | |  |
| **SIMD** |  |  |  |
| Low | 1444 | 128 | 0.09 |
| High | 1670 | 159 |  |
| Unknown | 104 | 16 |  |
|  | | |  |
| **Kidney Disease Status** | |  |  |
| Normal Kidney function | 1680 | 150 | 0.3 |
| Chronic Kidney disease | 1430 | 146 |  |
| Unknown | 108 | 7 |  |
|  | | |  |
| **SSRIs** |  |  |  |
| No SSRI | 2545 | 231 | 0.2 |
| Received an SSRI | 673 | 72 |  |
|  | | | |
| **Letrozole** | | | |
| No | 3167 | 297 | 0.7 |
| Yes | 51 | 6 |  |
|  | | | |
| **Surgery** | | | |
| No | 236 | 7 | P < 0.001* |
| Yes | 2982 | 296 |  |
|  | | | |
| **Chemotherapy** | | | |
| No | 2496 | 251 | P = 0.03* |
| Yes | 722 | 52 |  |
|  | | | |
| **Radiotherapy** | | | |
| No | 1027 | 73 | P = 0.03* |
| Yes | 2191 | 230 |  |

SIMD: Scottish Index of Multiple deprivation. SSRIs: selective serotonin reuptake inhibitors.

Table 2: Distribution of CYP2D6*4 allele across the cohorts

| **Cohort**  ***CYP2D6*4* genotype** | **GoSHARE** | **GoDARTS** | **GS:SFHS** | **Total** |
| --- | --- | --- | --- | --- |
| No **4* allele | 110 | 44 | 45 | 199 |
| Heterozygous | 45 | 28 | 22 | 95 |
| Homozygous | 4 | 4 | 1 | 9 |
| **Total** | 159 | 76 | 68* | **303** |

**Nine patients were reported in the GoSHARE or GoDARTS cohorts and were excluded from the GS:SFHS cohort count to avoid duplication.*

Table 3: Genotyped subpopulation and Fishers exact test

|  | **CYP2D6*4 null** | **Heterozygous** | **Homozygous** | **Fisher’s exact test P-value** |
| --- | --- | --- | --- | --- |
| Covariate | Median (range)/ No. of patients | Median (range)/ No. of patients | Median (range)/ No. of patients |  |
|  | | | | |
| **Age (categorical)** | | | | |
| **≤55** | 74 | 35 | 3 | 1 |
| **>55** | 125 | 60 | 6 |  |
|  | | | | |
| **Median follow-up time** (years) | 10.3 (0.05 – 21.4) | 8.6 (0.07 – 21.1) | 7.6 (0.06 – 21.4) | - |
|  | | | | |
| **Tumour size cm** | | | | |
| ≤2 | 128 | 65 | 5 | 0.45 |
| >2 | 64 | 25 | 3 |  |
| Unknown | 7 | 5 | 1 |  |
|  | | | | |
| **Tumour Grade** | | | | |
| Low | 33 | 22 | 0 | 0.23 |
| Intermediate | 125 | 48 | 8 |  |
| High | 39 | 24 | 1 |  |
| Unknown | 2 | 1 | 0 |  |
|  | | | | |
| **Lymph Nodes positive** | |  |  |  |
| No | 155 | 74 | 8 | 0.82 |
| Yes | 38 | 16 | 1 |  |
| Unknown | 6 | 5 | 0 |  |
|  | | | | |
| **Oestrogen Receptors positive** | |  |  |  |
| No | 4 | 3 | 0 | 0.75 |
| Yes | 195 | 92 | 9 |  |
|  | | | | |
| **SIMD** |  |  |  |  |
| Low | 81 | 42 | 5 | 0.88 |
| High | 106 | 49 | 4 |  |
| Unknown | 12 | 4 | 0 |  |
|  | | | | |
| **Kidney Disease Status** | |  |  |  |
| Normal Kidney function | 104 | 42 | 4 | 0.65 |
| Chronic Kidney disease | 90 | 51 | 5 |  |
| Unknown | 5 | 2 | 0 |  |
|  | | | | |
| **SSRIs** |  |  |  |  |
| No SSRI | 146 | 77 | 8 | 0.3 |
| Received an SSRI | 53 | 18 | 1 |  |
|  | | | | |
| **Letrozole** | | | | |
| No | 194 | 94 | 9 | 0.72 |
| Yes | 5 | 1 | 0 |  |
|  | | | | |
| **Surgery** | | | | |
| No | 3 | 4 | 0 | 0.37 |
| Yes | 196 | 91 | 9 |  |
|  | | | | |
| **Chemotherapy** | | | | |
| No | 161 | 83 | 7 | 0.28 |
| Yes | 38 | 12 | 2 |  |
|  | | | | |
| **Radiotherapy** | | | | |
| No | 47 | 22 | 4 | 0.37 |
| Yes | 152 | 73 | 5 |  |

SIMD: Scottish Index of Multiple deprivation. SSRIs: selective serotonin reuptake inhibitors.

*Table 4:* Univariate Cox Model for the whole cohort

|  | | **BCS** | | **OS** | | |
| --- | --- | --- | --- | --- | --- | --- |
| **Covariate** | | **HR (95% CI)** | **P-Value** | **HR (95% CI)** | **P-Value** | |
| **Tamoxifen quantity*** | | 0.980 (0.978– 0.982) | < 0.001* | 0.983 (0.982– 0.985) | < 0.001* | |
|  | | | | | | |
| **SSRIs Status*** | | | | | | |
| No SSRIs | | Reference |  |  |  | |
| Weak *CYP2D6* Inhibitor | | 0.46 (0.25 – 0.84) | 0.01* | 0.63 (0.44 – 0.92) | P = 0.02* | |
| Strong *CYP2D6* Inhibitor | | 0.39 (0.12 – 1.2) | 0.1 | 0.28 (0.1 – 0.75) | P = 0.01* | |
|  | | | | | | |
| **Age** | | | | | | |
| ≤50 | | Reference |  | Reference |  | |
| >50 | | 1.03 (0.85 – 1.26) | 0.74 | 2.73 (2.35 – 3.19) | P <0.001* | |
|  | | | | | | |
| **SIMD** | | | | | | |
| Low | | Reference |  | Reference |  | |
| High | | 0.78 (0.64 – 0.95) | P = 0.02* | 0.79 (0.70– 0.90) | P< 0.001* | |
| Unknown | | 0.61 (0.31 – 1.19) | P = 0.15 | 0.59 (0.38 – 0.91) | P = 0.02* | |
|  | | | | | | |
| **Tumour size** | |  |  |  |  | |
| ≤2 | | Reference |  | Reference |  | |
| >2 | | 5.06 (3.89 – 6.59) | P <0.001* | 2.06 (1.78 – 2.38) | P <0.001* | |
| Unknown | | 22.03 (16.5 – 29.42) | P <0.001* | 11.33 (9.58 – 13.39) | P <0.001* | |
|  | | | | | | |
| **Tumour Grade** | |  |  |  |  | |
| Low | | Reference |  | Reference |  | |
| Intermediate | | 3.23 (1.93 – 5.4) | P <0.001* | 1.42 (1.15 – 1.74) | P <0.001* | |
| High | | 8.5 (5.07 – 14.14) | P <0.001* | 2.05 (1.65 – 2.56) | P <0.001* | |
| Unknown | | 37.25 (21.28 – 65.21) | P <0.001* | 11.99 (9.2 – 15.62) | P <0.001* | |
|  | | | | | | |
| **Lymph Nodes** | |  |  |  |  | |
| No | | Reference |  | Reference |  | |
| Yes | | 6.12 (4.76 – 7.86) | P <0.001* | 1.99 (1.73 – 2.31) | P <0.001* | |
| Unknown | | 24.34 (18.31 – 32.22) | P <0.001* | 12.46 (10.55 – 14.72) | P <0.001* | |
|  | | | | | | |
| **Positive Oestrogen Receptors** | |  |  |  |  | |
| Yes | | Reference |  | Reference |  | |
| No | | 5.42 (4.08 – 7.19) | P <0.001* | 4.55 (3.74 – 5.52) | P <0.001* | |
|  | | | | | | |
| **Kidney Disease Status** | | | | | | |
| Normal Kidney function | | Reference |  | Reference |  | |
| Chronic Kidney disease | | 1.11 (0.91 – 1.36) | P = 0.29 | 2.26 (1.98 – 2.58) | P <0.001* | |
| Unknown | | 0.72 (0.37 – 1.39) | P = 0.33 | 1.43 (0.98 – 2.09) | P = 0.06 | |
|  | | | | | | |
| **Surgery** | | | | | | |
| No | Reference | |  | Reference | |  |
| Yes | 0.07 (0.05-0.08) | | P <0.001* | 0.07 (0.06-0.08) | | P <0.001* |
|  | | | | | | |
| **Chemotherapy** | | | | | | |
| No | | Reference |  | Reference |  | |
| Yes | | 2.58 (2.11 – 3.14) | P <0.001* | 0.92 (0.79 – 1.07) | P = 0.3 | |
|  | | | | | | |
| **Radiotherapy** | | | | | | |
| No | | Reference |  | Reference |  | |
| Yes | | 0.56 (0.46 – 0.69) | P <0.001* | 0.45 (0.4 – 0.51) | P <0.001* | |
|  | | | | | | |
| **Letrozole** | | | | | | |
| No | | Reference |  | Reference |  | |
| Yes | | 1.03 (0.46 – 2.31) | 0.94 | 1.45 (0.91 – 2.31) | P = 0.12 | |

The Univariate model: unadjusted Cox proportional hazard model. BCS: breast cancer specific survival. OS: overall survival. Tamoxifen Coverage: Tamoxifen quantity: the total number of tamoxifen standard daily doses in the prior six months as a time-dependent variable. SSRIs Status: categorical variable as time-dependent variable SIMD: Scottish Index of Multiple deprivation.

*Note: For the tamoxifen quantity, we used three decimal places because the HR was very narrow.*

Table 5: Univariate Cox Model (Genotyped cohort)

|  | **BCS** | | **OS** | |
| --- | --- | --- | --- | --- |
| **Covariate** | **HR (95% CI)** | **P-Value** | **HR (95% CI)** | **P-Value** |
| ***CYP2D6** Status** |  | | | |
| No **4* | Reference |  | Reference |  |
| Heterozygous | 3.18 (1.28 – 7.9) | 0.01* | 1.59 (0.98 – 2.57) | 0.06 |
| Homozygous | 3.39 (0.42 – 27.15) | 0.25 | 1.38 (0.34 – 5.72) | 0.65 |
|  | | | | |
| **Age** | | | | |
| ≤50 | Reference |  | Reference |  |
| >50 | 0.68 (0.28 – 1.64) | 0.4 | 2.45 (1.42 – 4.22) | P = 0.001* |
|  | | | | |
| **Tumour size** |  |  |  |  |
| ≤2 | Reference |  | Reference |  |
| >2 | 5.03 (1.58 – 16.05) | P = 0.01* | 1.9 (1.17 – 3.07) | P = 0.01* |
| Unknown | 65.26 (17.64 – 241.48) | P <0.001* | 12.23 (4.94 – 30.25) | P <0.001* |
|  | | | | |
| **Lymph Nodes** |  |  |  |  |
| No | Reference |  | Reference |  |
| Yes | 5.39 (1.87-15.55) | P = 0.002* | 1.74 (1.03 – 2.95) | P = 0.04* |
| Unknown | 49.66 (15.7-157.07) | P <0.001* | 9.92 (4.42 – 22.27) | P <0.001* |
|  | | | | |
| **Tumour Grade** | | | | |
| Low/intermediate | Reference |  | Reference |  |
| High | 5.6 (2.25 - 13.94) | P < 0.001* | 1.58 (0.94 - 2.66) | P = 0.08 |
| Unknown | 14.8 (1.84 – 119.11) | P = 0.01* | 2.18 (0.3 – 15.79) | P = 0.44 |

The Univariate model: unadjusted Cox proportional hazard model. BCS: breast cancer specific survival. OS: overall survival.

Table 6: Multivariable Cox Model (Genotyped cohort)

|  | **BCS** | | **OS** | |
| --- | --- | --- | --- | --- |
| **Covariate** | **HR (95% CI)** | **P-Value** | **HR (95% CI)** | **P-Value** |
| ***CYP2D6*4* Status** |  | | | |
| No **4* | Reference |  | Reference |  |
| Heterozygous | 3.7 (1.32 - 10.6) | 0.01* | 1.76 (1.07 - 2.9) | 0.025* |
| Homozygous | 11.6 (1.3 - 103.5) | 0.03* | 2.36 (0.56 - 10) | 0.24 |
|  | | | | |
| **Age** | | | | |
| ≤50 | Reference |  | Reference |  |
| >50 | 2.9 (0.99 - 8.3) | 0.051 | 4.69 (2.57-8.6) | P < 0.001* |
|  | | | | |
| **Tumour size** |  |  |  |  |
| ≤2 | Reference |  | Reference |  |
| >2 | 3 (0.8 - 11.6) | P = 0.1 | 2.14 (1.24 - 3.7) | P = 0.02* |
| Unknown | 16.6 (1.6 - 171.7) | P = 0.02* | 6.69 (1.49 - 30.1) | P = 0.01* |
|  | | | | |
| **Lymph Nodes** |  |  |  |  |
| No | Reference |  | Reference |  |
| Yes | 4.4 (1.34 - 14.8) | P = 0.02* | 1.69 (0.95 - 3) | P = 0.07 |
| Unknown | 21.8 (2.13 - 222) | P = 0.01* | 9.79 (2.27-42.2) | P = 0.002* |
|  | | | | |
| **Tumour Grade** | | | | |
| Low/intermediate | Reference |  | Reference |  |
| High | 7.6 (2.52 - 22.8) | P = 0.001* | 2.16 (1.23 – 3.8) | P = 0.02* |
| Unknown | 1.3 (0.12 - 15.1) | P = 0.81 | 0.51 (0.05 - 5.2) | P = 0.57 |

References

1. Abrahamowicz M, Beauchamp M-E, Sylvestre M-P. Comparison of alternative models for linking drug exposure with adverse effects. Statistics in Medicine. 2012;31(11-12):1014-30.
